# Supplementary material for: Enhanced β-adrenergic response in mice with dominant-negative expression of the PKD2L1 channel
Source: PLoS One. 2022 Jan 20;17(1):e0261668. doi: 10.1371/journal.pone.0261668 (PMC8775249; doi:10.1371/journal.pone.0261668)

Supporting Information

**Table S1**

**Oligo DNAs used in RT-PCR analyses**


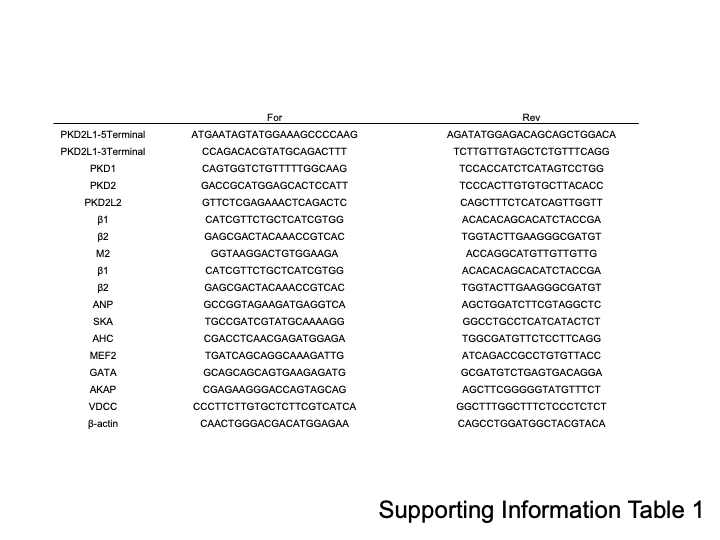


**Fig. S1**

**RT-PCR analysis**

1. RT-PCR data from hearts collected from WT and Tg mice. The primer sets used for PCR amplification are shown. Expression of β-actin was evaluated as a control.
2. Detection of endogenous PKD2L1 expression (507 bp) and transgene expression (235 bp) in the heart by RT-PCR. Specific primer set (PKD2L1-3Terminal) to distinguish the endogenous PKD2L1 gene.


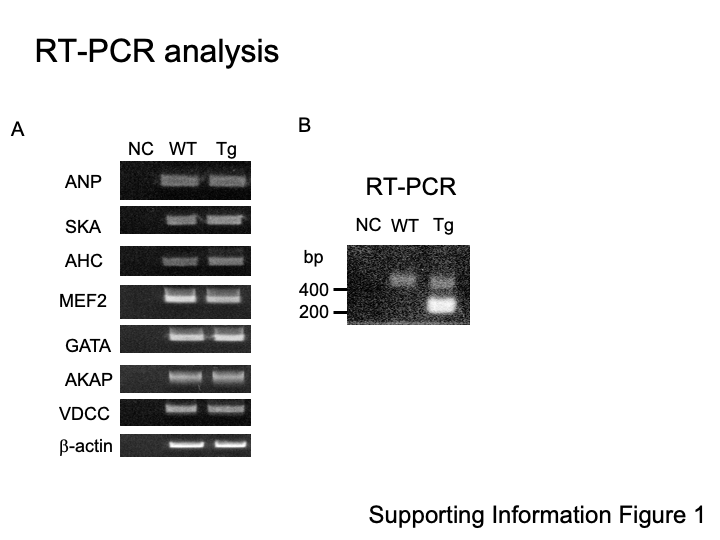


**Fig.S2**

**Enhanced response to catecholamine**

Increased contractility in PKD2L1del-Tg atria in response to norepinephrine (A) and epinephrine (B).

Dose-dependent changes in atrial contractility in response to norepinephrine (10–100 nM) and epinephrine (10–100 nM) in the WT and Tg mice. Error bars indicate the standard error. *N* = 6–7. **P* < 0.05, between atria from WT and Tg mice.

**
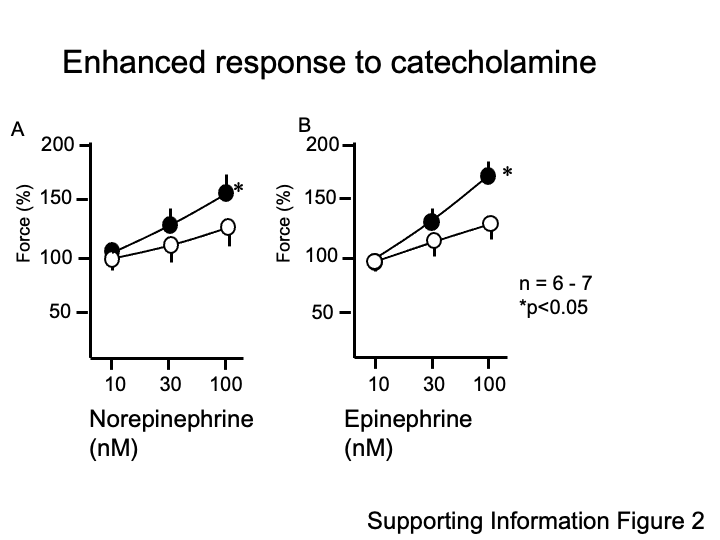
**

**Fig. S3**

Putative models of sympathetic stimulation in wild-type hearts (A) and enhanced cardiac response in PKD2L1del-Tg (B) hearts.

1. Wild-type PKD2L1 molecules associated with the cardiac β-receptor on the cell membrane. Once sympathetic tonus is elevated, norepinephrine (NE) binds to the β-receptor, resulting in increased cAMP and PKA activation.
2. In PKD2L1del-Tg hearts, an increased quantity of PKD2L1 without pore-forming domain molecules (ΔPKD2L1) form either a heteroTRPP3 channel (probably a non-functioning channel) or homo-ΔPKD2L1 channel (without a channel pore). Some endogenous wild-type PKD2L1 molecules also form normal PKD2L1 channels. All three types of PKD2L1 channel are associated with β-receptors, resulting in increased β-receptor population. In this condition, once sympathetic tone is activated, NE binds to the β-receptor, resulting in increased cAMP (as a second messenger) and PKA activity, followed by enhanced cardiac responses.

**
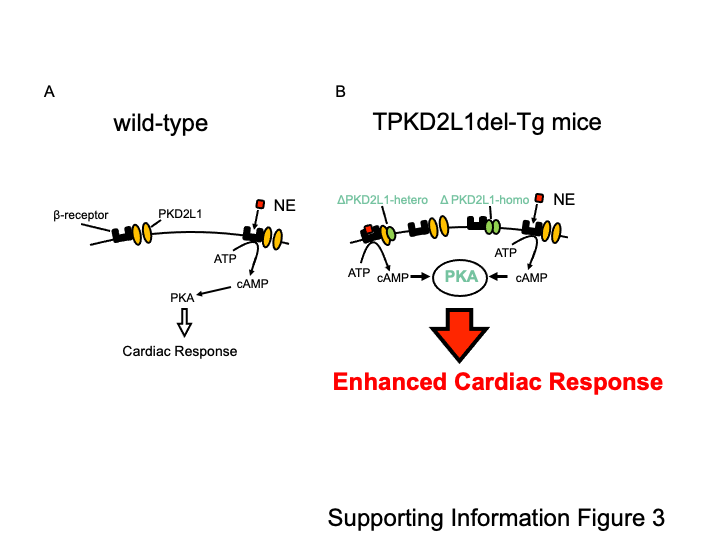
**

**Fig. S4**

1. PCR of PKD2L1-transgene in Tg-A (shaded bar) and Tg-B (closed bar). **P* < 0.05, between Tg-A and Tg-B. Each group consisted of six samples.
2. RT-PCR of the PKD2L1-transgene in Tg-A (shaded bars) and Tg-B (closed bars) with a specific primer set from Fig. 1B. **P* < 0.05, between Tg-A and Tg-B. Each group consisted of six samples.
3. RT-PCR of PKD2L1 in the WT (open bars) and Tg mice (closed bars) with a specific primer set from Fig. 1B in the kidney and heart. **P* < 0.05, between WT and Tg mice. Each group consisted of six samples.
4. RT-PCR of the adrenergic β1 receptor (β1R) and β2 receptor (β2R) in WT (open bars) and Tg mice (closed bars) in the heart. β-actin expression was examined as a control. **P* < 0.05, between WT and Tg mice. Each group consisted of six samples.


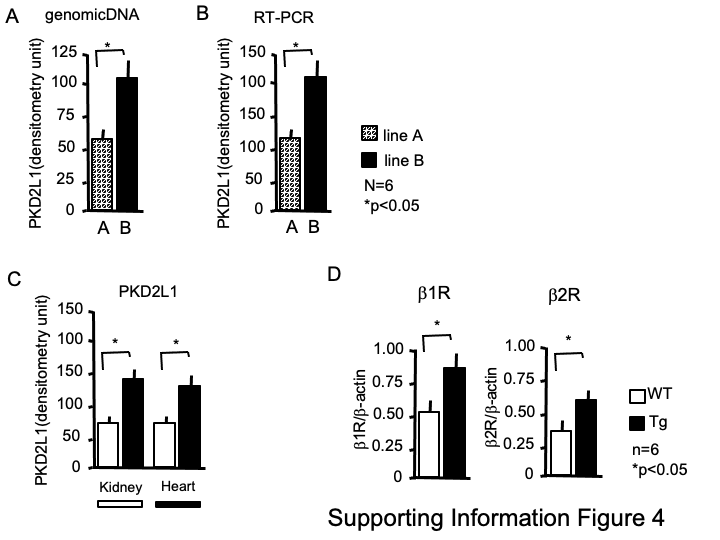


**Fig. S5**

1. **Original RT-PCR gels**

Primer sets are indicated.

1. **Original western blots**

The proteins examined are indicated.


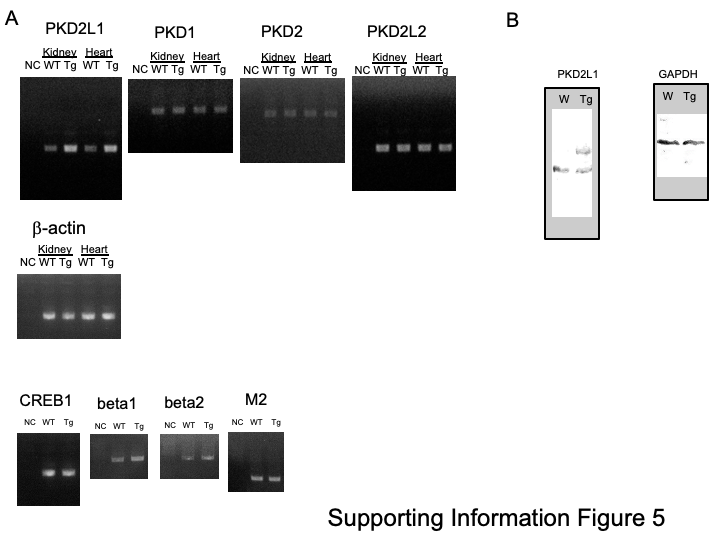


**SI The minimal data set**

Minimal data set of Fig. 2, Fig.3, Fig.4, and Fig. 5 graphs.


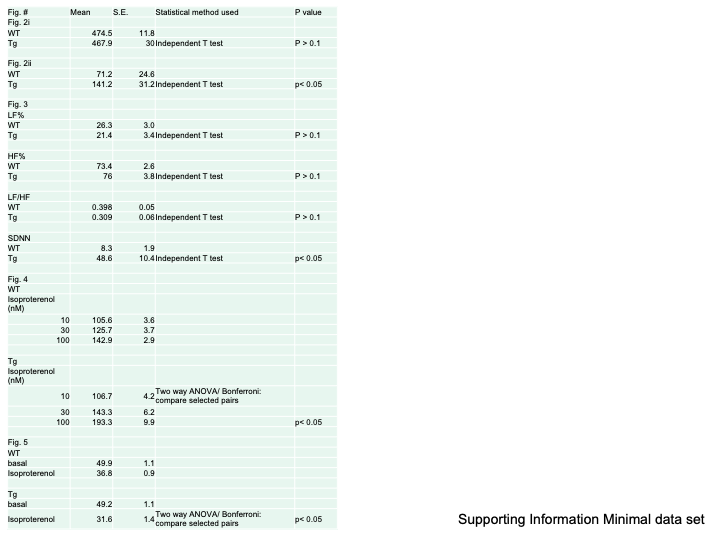

Supplement: S1 File — (DOCX) [file pone.0261668.s001.docx]
