# Supplementary figures and images for: Enhanced β-adrenergic response in mice with dominant-negative expression of the PKD2L1 channel
Source: PLoS One. 2022 Jan 20;17(1):e0261668. doi: 10.1371/journal.pone.0261668 (PMC8775249; doi:10.1371/journal.pone.0261668)

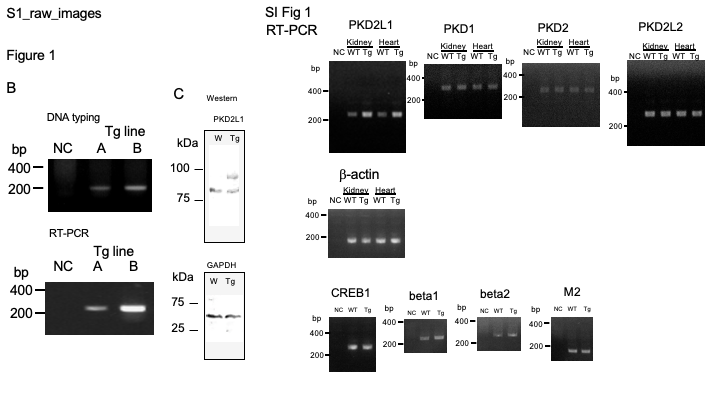

Supplement: S1 Raw images — (TIFF) [file pone.0261668.s002.tiff]
