# Supplementary material for: Enhanced β-adrenergic response in mice with dominant-negative expression of the PKD2L1 channel
Source: PLoS One. 2022 Jan 20;17(1):e0261668. doi: 10.1371/journal.pone.0261668 (PMC8775249; doi:10.1371/journal.pone.0261668)
Supplement: Attachment — Submitted filename: response letter Tg.docx [file pone.0261668.s003.docx]

Letter from *PLOS ONE* (*italic*)

(Our responses are in red)

*PONE-D-21-08901
Enhanced β-adrenergic response in mice with dominant-negative expression of the PKD2L1 channel
PLOS ONE

Dear Dr. Murakami,

Thank you for submitting your manuscript to PLOS ONE. After careful consideration, we feel that it has merit but does not fully meet PLOS ONE’s publication criteria as it currently stands. Therefore, we invite you to submit a revised version of the manuscript that addresses the points raised during the review process. While addressing the reviewer's concerns, please better uncropped images of Western blots. Also, better PCR images are needed. All bar graphs should be converted to dot plots with indicated mean and error bars.*

*Please submit your revised manuscript by Aug 07 2021 11:59PM. If you will need more time than this to complete your revisions, please reply to this message or contact the journal office at plosone@plos.org. When you're ready to submit your revision, log on to https://www.editorialmanager.com/pone/ and select the 'Submissions Needing Revision' folder to locate your manuscript file.*

*Please include the following items when submitting your revised manuscript:*

- *A rebuttal letter that responds to each point raised by the academic editor and reviewer(s). You should upload this letter as a separate file labeled 'Response to Reviewers'.*
- *A marked-up copy of your manuscript that highlights changes made to the original version. You should upload this as a separate file labeled 'Revised Manuscript with Track Changes'.*
- *An unmarked version of your revised paper without tracked changes. You should upload this as a separate file labeled 'Manuscript'.*

*If you would like to make changes to your financial disclosure, please include your updated statement in your cover letter. Guidelines for resubmitting your figure files are available below the reviewer comments at the end of this letter.

If applicable, we recommend that you deposit your laboratory protocols in protocols.io to enhance the reproducibility of your results. Protocols.io assigns your protocol its own identifier (DOI) so that it can be cited independently in the future. For instructions see:* [*http://journals.plos.org/plosone/s/submission-guidelines#loc-laboratory-protocols*](http://journals.plos.org/plosone/s/submission-guidelines#loc-laboratory-protocols)*. Additionally, PLOS ONE offers an option for publishing peer-reviewed Lab Protocol articles, which describe protocols hosted on protocols.io. Read more information on sharing protocols at* [*https://plos.org/protocols?utm_medium=editorial-email&utm_source=authorletters&utm_campaign=protocols*](https://plos.org/protocols?utm_medium=editorial-email&utm_source=authorletters&utm_campaign=protocols)*.

We look forward to receiving your revised manuscript.

Kind regards,

Alexander G Obukhov, Ph.D.
Academic Editor
PLOS ONE

Journal Requirements:*

*When submitting your revision, we need you to address these additional requirements.*

*1.Please ensure that your manuscript meets PLOS ONE's style requirements, including those for file naming. The PLOS ONE style templates can be found at*

*https://journals.plos.org/plosone/s/file?id=wjVg/PLOSOne_formatting_sample_main_body.pdf and*

[*https://journals.plos.org/plosone/s/file?id=ba62/PLOSOne_formatting_sample_title_authors_affiliations.pdf*](about:blank)

*>>>>*

We have ensured that the revised manuscript conforms to the style requirements of *PLOS ONE*. The authors have converted the bar graphs into bar graphs (means ± standard errors) with dots.

*2. To comply with PLOS ONE submissions requirements, please provide methods of sacrifice in the Methods section of your manuscript.*

*>>>>*

The manuscript has been revised in accordance with the Supporting Information guidelines. Additional information has been provided regarding compliance with the regulations for animal experiments.

In Methods:

All procedures were performed under general anesthesia as described below, and all efforts were made to minimize suffering. To isolate the heart, the mice were anesthetized with an intraperitoneal injection of a mixture of medetomidine hydrochloride (0.315 mg/kg), midazolam (2.0 mg/kg), and butorphanol tartrate (2.5 mg/kg). To prevent any pain associated with the injections, the mice were first anesthetized by inhaling 80% carbon dioxide and 20% oxygen prior to the intraperitoneal injection.

*3. PLOS ONE now requires that authors provide the original uncropped and unadjusted images underlying all blot or gel results reported in a submission’s figures or Supporting Information files. This policy and the journal’s other requirements for blot/gel reporting and figure preparation are described in detail at https://journals.plos.org/plosone/s/figures#loc-blot-and-gel-reporting-requirements and https://journals.plos.org/plosone/s/figures#loc-preparing-figures-from-image-files. When you submit your revised manuscript, please ensure that your figures adhere fully to these guidelines and provide the original underlying images for all blot or gel data reported in your submission. See the following link for instructions on providing the original image data: https://journals.plos.org/plosone/s/figures#loc-original-images-for-blots-and-gels.*

*In your cover letter, please note whether your blot/gel image data are in Supporting Information or posted at a public data repository, provide the repository URL if relevant, and provide specific details as to which raw blot/gel images, if any, are not available. Email us at plosone@plos.org if you have any questions.*

*>>>>>>>*

The original uncropped and unadjusted images of the DNA-typing and RT-PCR gels, and the western blots are presented in our Supporting Information Fig. 4.

The western blots in Fig. 1A (for PKD2L1 and GAPDH) were cut from original transferred membranes and subjected to immunoreaction because of the relatively low antibody dilutions (200–500-fold). Therefore, they are the original blots but are small.

*4. Thank you for stating the following in the Acknowledgments Section of your manuscript:*

*This research was sponsored in part by Grants-in-Aid for Scientific Research from JSPS, KAKENHI (17K08527, 17H04319, 16K09489, and 20K07255).*

*We note that you have provided funding information that is not currently declared in your Funding Statement. However, funding information should not appear in the Acknowledgments section or other areas of your manuscript. We will only publish funding information present in the Funding Statement section of the online submission form.*

*Please remove any funding-related text from the manuscript and let us know how you would like to update your Funding Statement. Currently, your Funding Statement reads as follows:*

*This research was sponsored in part by Grants-in-Aid for Scientific Research from JSPS, KAKENHI (17K08527, 17H04319, 16K09489, and 20K07255).*

*Please include your amended statements within your cover letter; we will change the online submission form on your behalf.*

>>>>>

The Funding Statement has been revised in accordance with the above guidelines:

This research was sponsored, in part, by Grants-in-Aid for Scientific Research from JSPS, KAKENHI (17K08527, 17H04319, 16K09489, and 20K07255). No additional external funding was received for this study.

*5. In your Data Availability statement, you have not specified where the minimal data set underlying the results described in your manuscript can be found. PLOS defines a study's minimal data set as the underlying data used to reach the conclusions drawn in the manuscript and any additional data required to replicate the reported study findings in their entirety. All PLOS journals require that the minimal data set be made fully available. For more information about our data policy, please see http://journals.plos.org/plosone/s/data-availability.*

*Upon re-submitting your revised manuscript, please upload your study’s minimal underlying data set as either Supporting Information files or to a stable, public repository and include the relevant URLs, DOIs, or accession numbers within your revised cover letter. For a list of acceptable repositories, please see http://journals.plos.org/plosone/s/data-availability#loc-recommended-repositories. Any potentially identifying patient information must be fully anonymized.*

*Important: If there are ethical or legal restrictions to sharing your data publicly, please explain these restrictions in detail. Please see our guidelines for more information on what we consider unacceptable restrictions to publicly sharing data: http://journals.plos.org/plosone/s/data-availability#loc-unacceptable-data-access-restrictions. Note that it is not acceptable for the authors to be the sole named individuals responsible for ensuring data access.*

*We will update your Data Availability statement to reflect the information you provide in your cover letter.*

*>>>>>>>*

A minimal data set is available in Supporting Information Minimal data set.

*6. Please upload a copy of Supporting Information Figures S1, S2, S3 and S4 which you refer to in your text on page 24 and 25.*

*>>>>>>>*

The authors have uploaded the Supporting Information and Figures S1, S2, S3, and S4.

*[Note: HTML markup is below. Please do not edit.]

Reviewers' comments:

Reviewer's Responses to Questions*

***Comments to the Author*** *1. Is the manuscript technically sound, and do the data support the conclusions?

The manuscript must describe a technically sound piece of scientific research with data that supports the conclusions. Experiments must have been conducted rigorously, with appropriate controls, replication, and sample sizes. The conclusions must be drawn appropriately based on the data presented.*

*Reviewer #1: Yes*

*2. Has the statistical analysis been performed appropriately and rigorously?*

*Reviewer #1: Yes*

*3. Have the authors made all data underlying the findings in their manuscript fully available?

The* [*PLOS Data policy*](http://www.plosone.org/static/policies.action#sharing) *requires authors to make all data underlying the findings described in their manuscript fully available without restriction, with rare exception (please refer to the Data Availability Statement in the manuscript PDF file). The data should be provided as part of the manuscript or its supporting information, or deposited to a public repository. For example, in addition to summary statistics, the data points behind means, medians and variance measures should be available. If there are restrictions on publicly sharing data—e.g. participant privacy or use of data from a third party—those must be specified.*

*Reviewer #1: Yes*

*4. Is the manuscript presented in an intelligible fashion and written in standard English?

PLOS ONE does not copyedit accepted manuscripts, so the language in submitted articles must be clear, correct, and unambiguous. Any typographical or grammatical errors should be corrected at revision, so please note any specific errors here.*

*Reviewer #1: Yes*

*5. Review Comments to the Author

Please use the space provided to explain your answers to the questions above. You may also include additional comments for the author, including concerns about dual publication, research ethics, or publication ethics. (Please upload your review as an attachment if it exceeds 20,000 characters)*

*Reviewer #1: Polycystic kidney disease 2-like 1 protein also known as transient receptor potential polycystic 2 (TRPP2) is a protein that in humans is encoded by the PKD2L1 gene. This study reports the development and characterisation of a transgenic mouse line overexpressing a dominant negative form of PKD2L1. The phenotype showed an augmented sensitivity to B-adrenergic stimulation, an unstable R-R interval and elevated paramertes of sympathetic regulation. The paper is generally well written and presented, and the data will be of interest to better understand the pathogenesis of ADPKD.

Comments
1. The Introduction seems dis-jointed. For example, the rationale and link with ADPKD is not clear and could be better explained
>>>>>*

The manuscript has been revised in accordance with the comments. The authors have revised the Introduction to explain the relationship between PKD and TRPP.

*2. As this is the first description of this mouse line, it would be important to report whether there any other phenotype, such as the histological presence of kidney cysts and/or vascular abnormalities*

*>>>>>*

Thank you for your comments. This point is important. The authors expected a hereditary dominant phenotype in the kidney, but the histological examination revealed no obvious changes in the heart or kidneys.

In the Results:

The histological examination revealed no obvious changes in the heart or kidneys.

*6. PLOS authors have the option to publish the peer review history of their article (*[*what does this mean?*](https://journals.plos.org/plosone/s/editorial-and-peer-review-process#loc-peer-review-history)*). If published, this will include your full peer review and any attached files.


If you choose “no”, your identity will remain anonymous but your review may still be made public.*


***Do you want your identity to be public for this peer review?*** *For information about this choice, including consent withdrawal, please see our* [*Privacy Policy*](https://www.plos.org/privacy-policy)*.*

*Reviewer #1: No*

*[NOTE: If reviewer comments were submitted as an attachment file, they will be attached to this email and accessible via the submission site. Please log into your account, locate the manuscript record, and check for the action link "View Attachments". If this link does not appear, there are no attachment files.]

While revising your submission, please upload your figure files to the Preflight Analysis and Conversion Engine (PACE) digital diagnostic tool,* [*https://pacev2.apexcovantage.com/*](https://pacev2.apexcovantage.com/)*. PACE helps ensure that figures meet PLOS requirements. To use PACE, you must first register as a user. Registration is free. Then, login and navigate to the UPLOAD tab, where you will find detailed instructions on how to use the tool. If you encounter any issues or have any questions when using PACE, please email PLOS at* [*figures@plos.org*](mailto:figures@plos.org)*. Please note that Supporting Information files do not need this step.*
