# Supplementary material for: Enhanced β-adrenergic response in mice with dominant-negative expression of the PKD2L1 channel
Source: PLoS One. 2022 Jan 20;17(1):e0261668. doi: 10.1371/journal.pone.0261668 (PMC8775249; doi:10.1371/journal.pone.0261668)
Supplement: Attachment — Submitted filename: RebuttalLetter2411.docx [file pone.0261668.s004.docx]

We thank the editor and reviewers for reviewing our manuscript and suggesting revisions. We have addressed all of the reviewers’ queries.

Our point-by-point responses to each of the comments are presented in this letter (Our responses are in red).

Sincerely yours,

Manabu Murakami

Letter from *PLOS ONE* (*italic*)

***PLOS ONE Decision: Revision required [PONE-D-21-08901R1] - [EMID:e454b98252479c05]***

***PO***

*PLOS ONE*

*Thu 10/21/2021 7:13 AM*

*CC: aobukhov@iupui.edu, alexanderobukhov@yahoo.com*

*PONE-D-21-08901R1*

*Enhanced β-adrenergic response in mice with dominant-negative expression of the PKD2L1 channel*

*PLOS ONE  Dear Dr. Murakami,  Thank you for submitting your manuscript to PLOS ONE. After careful consideration, we feel that it has merit but requires some minor revisions before it can be accepted for publication in PLOS ONE. Therefore, we invite you to submit a revised version of the manuscript that addresses the points raised by the reviewer.*

*Please submit your revised manuscript by Dec 04 2021 11:59PM. If you will need more time than this to complete your revisions, please reply to this message or contact the journal office at* [*plosone@plos.org*](mailto:plosone@plos.org%22%20%5Ct%20%22_blank)*. When you're ready to submit your revision, log on to https://www.editorialmanager.com/pone/ and select the 'Submissions Needing Revision' folder to locate your manuscript file.  Please include the following items when submitting your revised manuscript:*

1994. *•	A rebuttal letter that responds to each point raised by the reviewer. You should upload this letter as a separate file labeled 'Response to Reviewers'.*
1995. *•	A marked-up copy of your manuscript that highlights changes made to the original version. You should upload this as a separate file labeled 'Revised Manuscript with Track Changes'.*
1996. *•	An unmarked version of your revised paper without tracked changes. You should upload this as a separate file labeled 'Manuscript'.*

*If you would like to make changes to your financial disclosure, please include your updated statement in your cover letter. Guidelines for resubmitting your figure files are available below the reviewer comments at the end of this letter.  If applicable, we recommend that you deposit your laboratory protocols in protocols.io to enhance the reproducibility of your results. Protocols.io assigns your protocol its own identifier (DOI) so that it can be cited independently in the future. For instructions see:* [*https://journals.plos.org/plosone/s/submission-guidelines#loc-laboratory-protocols*](https://journals.plos.org/plosone/s/submission-guideli)*. Additionally, PLOS ONE offers an option for publishing peer-reviewed Lab Protocol articles, which describe protocols hosted on protocols.io. Read more information on sharing protocols at* [*https://plos.org/protocols?utm_medium=editorial-email&utm_source=authorletters&utm_campaign=protocols*](https://plos.org/protocols?utm_medium=editorial-email&utm_source=authorletters&utm_campaign=protocols%22%20%5Ct%20%22_blank)*.*

*We look forward to receiving your revised manuscript.*

*Kind regards,*

*Alexander G Obukhov, Ph.D.*

*Academic Editor*

*PLOS ONE*

*Journal Requirements:  Please review your reference list to ensure that it is complete and correct. If you have cited papers that have been retracted, please include the rationale for doing so in the manuscript text, or remove these references and replace them with relevant current references. Any changes to the reference list should be mentioned in the rebuttal letter that accompanies your revised manuscript. If you need to cite a retracted article, indicate the article’s retracted status in the References list and also include a citation and full reference for the retraction notice.*

*[Note: HTML markup is below. Please do not edit.]*

*Reviewers' comments:*

*Reviewer's Responses to Questions*

***Comments to the Author****1. If the authors have adequately addressed your comments raised in a previous round of review and you feel that this manuscript is now acceptable for publication, you may indicate that here to bypass the “Comments to the Author” section, enter your conflict of interest statement in the “Confidential to Editor” section, and submit your "Accept" recommendation.*

*Reviewer #2: All comments have been addressed*

*2. Is the manuscript technically sound, and do the data support the conclusions?  The manuscript must describe a technically sound piece of scientific research with data that supports the conclusions. Experiments must have been conducted rigorously, with appropriate controls, replication, and sample sizes. The conclusions must be drawn appropriately based on the data presented.*

*Reviewer #2: Yes*

*3. Has the statistical analysis been performed appropriately and rigorously?*

*Reviewer #2: I Don't Know*

*4. Have the authors made all data underlying the findings in their manuscript fully available?  The* [*PLOS Data policy*](http://www.plosone.org/static/policies.action%22%20%5Cl%20%22sharing%22%20%5Ct%20%22_blank) *requires authors to make all data underlying the findings described in their manuscript fully available without restriction, with rare exception (please refer to the Data Availability Statement in the manuscript PDF file). The data should be provided as part of the manuscript or its supporting information, or deposited to a public repository. For example, in addition to summary statistics, the data points behind means, medians and variance measures should be available. If there are restrictions on publicly sharing data—e.g. participant privacy or use of data from a third party—those must be specified.*

*Reviewer #2: Yes*

*5. Is the manuscript presented in an intelligible fashion and written in standard English?  PLOS ONE does not copyedit accepted manuscripts, so the language in submitted articles must be clear, correct, and unambiguous. Any typographical or grammatical errors should be corrected at revision, so please note any specific errors here.*

*Reviewer #2: Yes*

*6. Review Comments to the Author*

*Please use the space provided to explain your answers to the questions above. You may also include additional comments for the author, including concerns about dual publication, research ethics, or publication ethics. (Please upload your review as an attachment if it exceeds 20,000 characters)*

*Reviewer #2: Murakami et al. created PKD2L1del-Tg mice to investigate physiological role of PKD2L1channel, particularly for β-adrenergic stimulation. Using this new model, the authors measured EKG, inotropic atrial contractions, and action potentials. Their measurements show that PKD2L1del-Tg mice have increased adrenergic sensitivity indicating PKD2L1 involvement to sympathetic regulation. The study is important and well-done. I only have a question about statistics and an optional suggestion to improve the paper Discussion.   Please clarify statistics: Figures 4 and 5: What is “n” in the figure legends: n =6~7 and n = 8-12. Is it number of animals, number of cells, or number of measurements?*

>>>>

Thank you for your comment. These experiments were conducted on cardiac tissues; therefore, “*n*” refers to the number of measurements.

Figure 4 shows the measurement of isolated left atrial contraction. Therefore, “*n*” refers to the number of measurements. A single left atrium was obtained from one mouse. Therefore, “*n*” was equivalent to the number of animals. We have explained the contraction analysis (inotropic atrial contraction) in the Methods section.

Figure 5 shows the action potential measured from the isolated right atrium. Therefore, “*n*” refers to the number of measurements, similar to Figure 4. A single right atrium was obtained from one mouse. Therefore, “*n*” also refers to number of animals. We have briefly explained the action potential analysis in the Methods section.

>>>>

*Discussion: PKD2L1 is a cation channel. However according to the literature and results of the present study, its involvement to sympathetic regulation may not be necessarily linked to its activity as a channel, but to its role in regulation of β2AR trafficking or some other activity. The paper discussion is rather short, and I would encourage the authors to write more about possible mechanisms of PKD2L1 involvement in sympathetic regulation. Based on the new results of the present study and literature data, please make a schematic figure to illustrate possible interactions and possible mechanisms of how PKD2L1 regulate β-adrenergic stimulation.*

*Line :370: “Additional analyses are needed to elucidate the precise mechanism of β2AR trafficking and the roles of PKD channels in controlling the cAMP signaling cascade.” Please specify what specific analyses and/or experiments are needed to clarify the mechanisms.*

>>>

Thank you for your important comment.

Although evidence regarding the underlying mechanism is not conclusive, we have constructed a diagram of the hypothesized underlying mechanism (Fig S3).

It is essential to analyze the possible mechanisms underlying enhanced cardiac responses to sympathetic nerve stimuli in PKD2L1del-Tg mice. However, we do not have enough data to construct a diagram. Therefore, we have designed a scheme to explain our hypotheses regarding the association of PKD2L1 molecules with β-receptors, and the effects of PKD2L1 receptors on cardiac responses (Fig S3).

The study conclusions may be verified by analyzing the levels of β-receptors, endogenous PKD2L1, other TRPP proteins, and cAMP, and the PKA-dependent phosphorylation of target molecules, such as CREB. To determine the mechanisms underlying enhanced sympathetic responses in PKD2L1del-Tg mice, the interaction between PKD2L1 and β-adrenergic receptors should be evaluated. This would involve analysis of the PKD2L1 ionic currents in the cardiac myocytes from wild-type and PKD2L1-delta transgenic mice.

The aforementioned experiments would require more than 1 year. We did not mention these cascades in the manuscript because we thought that this might have prompted the reviewers to ask us to perform these experiments.

However, we have added a diagram (Fig S3) and additional text to the Discussion section.

>>>

*7. PLOS authors have the option to publish the peer review history of their article (*[*what does this mean?*](https://journals.plos.org/plosone/s/editorial-an)*). If published, this will include your full peer review and any attached files.   If you choose “no”, your identity will remain anonymous but your review may still be made public.****Do you want your identity to be public for this peer review?*** *For information about this choice, including consent withdrawal, please see our* [*Privacy Policy*](https://www.plos.org/privacy-policy%22%20%5Ct%20%22_blank)*.*

*Reviewer #2: No*

*[NOTE: If reviewer comments were submitted as an attachment file, they will be attached to this email and accessible via the submission site. Please log into your account, locate the manuscript record, and check for the action link "View Attachments". If this link does not appear, there are no attachment files.]  While revising your submission, please upload your figure files to the Preflight Analysis and Conversion Engine (PACE) digital diagnostic tool,* [*https://pacev2.apexcovantage.com/*](https://pacev2.apexcovantage.com/)*. PACE helps ensure that figures meet PLOS requirements. To use PACE, you must first register as a user. Registration is free. Then, login and navigate to the UPLOAD tab, where you will find detailed instructions on how to use the tool. If you encounter any issues or have any questions when using*
